# Anti-tubercular derivatives of rhein require activation by the monoglyceride lipase Rv0183

**DOI:** 10.1016/j.tcsw.2020.100040

**Published:** 2020-04-21

**Authors:** Katherine A. Abrahams, Wei Hu, Gang Li, Yu Lu, Emily J. Richardson, Nicholas J. Loman, Haihong Huang, Gurdyal S. Besra

**Affiliations:** a Institute of Microbiology and Infection, School of Biosciences, University of Birmingham, Edgbaston, Birmingham B15 2TT, UK; b State Key Laboratory of Bioactive Substances and Function of Natural Medicine, Beijing Key Laboratory of Active Substance Discovery and Druggability Evaluation, Institute of Materia Medica, Peking Union Medical College and Chinese Academy of Medical Sciences, 1 Xian Nong Tan Street, Beijing 100050, China; c Beijing Key Laboratory of Drug Resistance Tuberculosis Research, Department of Pharmacology, Beijing Tuberculosis and Thoracic Tumor Research Institute, Beijing Chest Hospital, Capital Medical University, 97 Ma Chang Street, Beijing 101149, China; d MicrobesNG, Units 1-2 First Floor, The BioHub, Birmingham Research Park, 97 Vincent Drive, Birmingham B15 2SQ, UK

**Keywords:** Rhein, Mycobacterium, Lipase, Drug discovery

## Abstract

The emergence and perseverance of drug resistant strains of *Mycobacterium tuberculosis* (*Mtb*) ensures that drug discovery efforts remain at the forefront of tuberculosis research. There are numerous different approaches that can be employed to lead to the discovery of anti-tubercular agents. In this work, we endeavored to optimize the anthraquinone chemical scaffold of a known drug, rhein, converting it from a compound with negligible activity against *Mtb*, to a series of compounds with potent activity. Two compounds exhibited low toxicity and good liver microsome stability and were further progressed in attempts to identify the biological target. Whole genome sequencing of resistant isolates revealed inactivating mutations in a monoglyceride lipase. Over-expression trials and an enzyme assay confirmed that the designed compounds are prodrugs, activated by the monoglyceride lipase. We propose that rhein is the active moiety of the novel compounds, which requires chemical modifications to enable access to the cell through the extensive cell wall structure. This work demonstrates that re-engineering of existing antimicrobial agents is a valid method in the development of new anti-tubercular compounds.

## 1 Introduction

*Mycobacterium tuberculosis* (*Mtb*), the etiological agent of tuberculosis (TB), is the ninth leading cause of death worldwide and is the primary cause of death from a single infectious agent. In 2018, an estimated 10 million people were diagnosed with the disease, which caused an estimated 1.4 million deaths. Efforts to reduce the incidence and mortality rates are being sought by the World Health Organization’s End TB Strategy and the United Nations’ Sustainable Development Goals, but it is apparent that progress is not sufficient to meet targets to ultimately end the global TB epidemic (World Health Organisation, 2019). The standard six-month treatment regimen for TB has remained unchanged over several decades, facilitating the development of drug resistance with an increasing prevalence of multi-drug resistant (MDR) and extensively-drug resistant (XDR) strains of *Mtb.* Currently there are 23 drugs in Phase I, II or III clinical trials, including new and repurposed drugs (World Health Organisation, 2019). In addition to early diagnosis, it is essential that new treatment strategies are developed to preserve the existing treatment program and to combat drug resistance.

Drug resistance can manifest by a number of different mechanisms including mutations to the target (Abrahams et al., 2016, Abrahams et al., 2012, Abrahams et al., 2017, Gurcha et al., 2014), transcriptional regulation (for example down-regulation of drug importers or up-regulation of exporters (Milano et al., 2009)), chemical modification (inactivating an inhibitor (Kasik, 1965) or preventing the conversion of a prodrug into an active compound (Rouse and Morris, 1995)). In order to establish the effectiveness of a drug, if possible, it is important to not only establish the target of the inhibitor but also to determine any associated resistance mechanisms that could be used to make the compound ineffective in a clinical setting. Thereby, decisions can be made as to whether to progress a compound in medicinal chemistry efforts or to employ alternative strategies to combat the resistance mechanism. For example, until recently, β-lactam antibiotics were considered to be ineffective against *Mtb* due to the expression of a β-lactamase. However, combination therapy with a β-lactam and a β-lactamase inhibitor has been shown to be bactericidal against replicating and non-replicating *Mtb* (Hugonnet et al., 2009, Rullas et al., 2015).

The continuing emergence of drug resistant strains of *Mtb* threatens to render current treatment programs ineffective, reinforcing the urgent requirement of new anti-tubercular agents. Innovative strategies are being sought to broaden the potential of developing or discovering the next front-line antibiotic. These include: re-purposing of existing approved drugs (Harbut et al., 2015, Maitra et al., 2015), such as known antibacterials; high-throughput phenotypic screening of extensive compound libraries (Ballell et al., 2013); target based screening (Moreira et al., 2015); optimizing the chemical scaffold of known drugs (Nigam et al., 2014). Given the global efforts in the search for new anti-tubercular agents, together with diversity of these approaches employed, it is anticipated that more compounds will make it into clinical trials for progression into the treatment regimen.

**Fig. 1:**
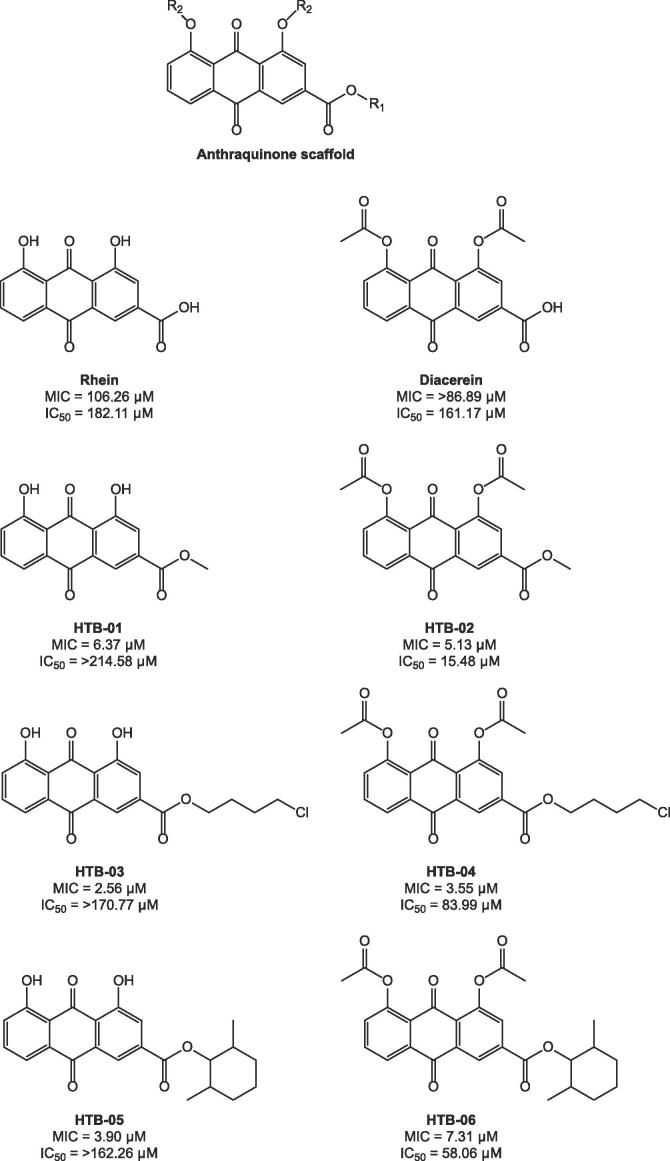
**Chemical structures of the anthraquinone rhein, diacerein and synthesized derivatives**. The *Mtb* MIC and cytotoxicity (Vero, IC_50_) of each compound is illustrated.

Re-purposing of existing antibacterial drug classes and structural modifications based on natural products with antibacterial activity are undoubtedly effective approaches to discover new anti-tubercular agents. In this work, we endeavored to optimize the chemical scaffold of the natural plant product rhein (Fig. 1). First isolated in 1895 from Chinese rhubarb, rhein has since been extracted from other plant species such as *Cassia reticulata* (Anchel, 1949). It has been shown to exhibit a multitude of medicinal properties including anti-cancer, anti-inflammatory, anti-diabetic and anti-microbial effects (Zhou et al., 2015). Consequently, rhein has an impact on a number of metabolic processes, examples of which are electron transport (Floridi et al., 1990), ATP production (Castiglione et al., 1990), and oxidative phosphorylation (Floridi et al., 1990) in eukaryotic cells. With relevance to its antimicrobial activity, rhein has been shown to inhibit *Helicobacter pylori* (Chung et al., 1998) and *Staphylococcus aureus* (Yu et al., 2008), although the mode of action is yet to be confirmed. Rhein does not exhibit anti-tubercular activity (Gajadeera et al., 2015). However, owing to the pharmacological potential rhein, we investigated whether structurally optimizing the anthraquinone core structure could convert an essentially inactive compound into a potent inhibitor against *Mtb*.

Herein, we show that the anthraquinone rhein and diacerein derivatives, HTB-03 and HTB-04, with a middle size 4-chloro-1-butyl ester group, demonstrated potent anti-TB activity, low toxicity and good liver microsome stability. Furthermore, we have determined that these compounds are prodrugs, which require activation by the monoglyceride lipase, Rv0183. *Mycobacterium bovis* BCG spontaneous resistant mutants raised against these compounds exhibit amino acid substitutions, deletion and STOP mutations in the Rv0183 homologue BCG_0220, thereby reducing or inactivating the enzyme activity, leading to the identification of a novel resistance mechanism. This is informative for the development of these compounds and for future hit optimization studies through focused medicinal chemistry efforts.

## 2 Materials and methods

### 2.1 HTB compounds

To a magnetically stirred solution of rhein/diacerein (0.2 mmol) in CH_2_Cl_2_ (2 mL), thionyl chloride (1 mL) was added. The reaction mixture was heated to reflux for 4 h under an atmosphere of argon. After cooling to room temperature, the reaction mixture was evaporated under reduced pressure. The residue was dissolved in anhydrous CH_2_Cl_2_ (4 mL) and 4-dimethylaminopyridine (DMAP) (0.04 mmol) was added and cooled with an ice bath. The mixture of selected alcohol (0.25 mmol) and CH_2_Cl_2_ (2 mL) were slowly added to the reaction mixture cooled with an ice bath. The reaction mixture was stirred for 3–6 h at room temperature. The solvent was evaporated under reduced pressure and the residue was purified by column chromatography (EtOAc: petroleum ether = 1:5) to give the title compounds. The general experimental information and structural characterization are provided in the Supplementary Material.

### 2.2 Minimum inhibitory concentration (MIC) and cytotoxicity assay

All target compounds were evaluated for their anti-mycobacterial activities against *Mtb* H_37_Rv or isolated clinical strains using the Microplate Alamar Blue Assay (MABA) (Collins and Franzblau, 1997). The MIC was defined as the lowest concentration effecting a reduction in fluorescence of ≥90% relative to the mean of replicate bacterium-only controls. All target compounds were further tested for their cytotoxicity against a mammalian cell line (Vero cells) using methyl-thiazolyldiphenyl-tetrazolium bromide (MTT) as a read out for cell viability and measured by the concentration required for inhibiting 50% cell growth (IC_50_) as compared to the no-treatment control (Lu et al., 2011).

### 2.3 Liver microsome stability assay

The assay was performed with liver microsomes from male CD-1 mouse (Xenotech) and pooled human (Bioreclamation). Compounds HTB-03, HTB-04 and HTB-05 were tested at 1 μM with a final concentration of microsomal protein of 1 mg/mL. The reaction was initiated by the addition of NADPH (1 mM), and samples were incubated for up to 60 min at 37 °C in a shaking incubator. The reaction was terminated at 0, 5, 15 and 30 min by the addition of ice-cold ACN/MeOH (50:50) spiked with internal standard. Samples were centrifuged at 4000 rpm at 4 °C for 15 min and the supernatants analyzed by LC-MS/MS. The assay evaluated the metabolic stability of compounds by measuring the amount of parent remaining to test compounds with or without NADPH cofactor. LC-MS/MS: AB Sciex 4500 Qtrap; Kinetex 2.6 μm C18 100 Å column (3.0 mm × 30 mm); mobile phase A, 0.1% formic acid in H_2_O; mobile phase B, 0.1% formic acid in ACN; flow rate, 0.8 mL/min; room temperature.

### 2.4 Culturing of mycobacteria

*M. bovis* BCG strain Pasteur and derivatives were cultured in static liquid media (Middlebrook 7H9 (Difco), 0.05% (v/v) Tween-80, 10% (v/v) Middlebrook ADC and 0.25% (v/v) glycerol) or on solid media (Middlebrook 7H11 agar (Difco) with 10% (v/v) Middlebrook OADC and 0.5% (v/v) glycerol) at 37 °C, 5% CO_2_. Where appropriate, 25 μg/mL kanamycin was added to the media to select for mycobacterial plasmids.

### 2.5 Generation of spontaneous resistant mutants and whole genome sequencing

*M. bovis* BCG was cultured to mid-log phase (OD_600nm_ 0.4–0.8) and 1 × 10^8^ cells were plated on solid media containing 50× and 100× MIC HTB-03 and 20× MIC HTB-04 (based on MICs for *M. tuberculosis* H_37_Rv). Potential resistant mutants were cultured and reselected on 100× MIC HTB-03 and 20× MIC HTB-04 to confirm the resistant phenotype. The genomic DNA (gDNA) from the mutants was purified following standard methods and the whole genome sequencing (WGS) and bioinformatics were performed by MicrobesNG, University of Birmingham. Briefly, barcoded DNA libraries were generated from genomic DNA, the fragments were purified and quantified before size evaluation and sequencing. High frequency, statistically relevant single nucleotide polymorphisms (SNPs) were identified by aligning the mutant sequence reads to the *M. bovis* BCG Pasteur 1173P2 (accession: NC_008769.1) genome sequence.

### 2.6 Preparation of plasmid expression constructs

The plasmids pVV16 and pET28a were used for over-expression studies in *M. bovis* BCG and *Escherichia coli* respectively, both of which encode kanamycin resistance for selection. Vector-encoded poly-histidine tags were exploited from both pVV16 (C-terminal) and pET28a (N-terminal). Primers were designed to clone WT *BCG_0220* (equivalent to *Rv0183* from *Mtb* H_37_Rv) and the mutant genes, *BCG_0220*-S172P and *BCG_0220*-VAAQD137D, from *M. bovis* BCG genomic DNA (isolated as described above) into the multiple cloning sites of the plasmids, exploiting the restriction sites *Nde*I and *Bam*HI, and are listed in Table 1. The *Rv0183* gene was amplified using Q5 DNA polymerase (New England Biolabs) following the manufacturer’s instructions. The PCR products were purified (QIAquick PCR Purification Kit, Qiagen), and along with the plasmids, digested with restriction enzymes (FastDigest, Thermo Fisher Scientific) and the products were extracted from a 1% agarose gel (QIAquick Gel Extraction Kit, Qiagen). The digested PCR products and vectors were ligated using T4 DNA ligase (New England Biolabs). Following transformation into *E. coli* TOP10 cells, the constructs in positive clones were confirmed by DNA sequencing (Source Bioscience).

**Table 1:** Primers used for the generation of over-expression constructs. The restriction site is identified in bold type.

Primer	Plasmid	Sequence (5′-3′)
Rv0183 sense	pVV16	GATCGACT**CATATG**ACTACCACCCGGACTGAACG
	pET28a	
Rv0183 Anti-sense	pVV16	GATCGATC**GGATCC**CCAACCGCTCGGTGAGCCAG
	pET28	GATCGATC**GGATCC**CTACAACCGCTCGGTGAGC

### 2.7 Electroporation of mycobacteria

Electrocompetent *M. bovis* BCG (HTB-04 mutant 1, BCG_0220-VAAQD137D and HTB-04 mutant 2, BCG_0220-S172P) were prepared by pelleting and subsequently washing a mid-log culture with decreasing volumes of room temperature 10% (v/v) glycerol. The cells were incubated at 37 °C with 1 μg plasmid DNA (pVV16 or pVV16-*Rv0183*) for 10 min in a 2 mm electrode gap electroporation cuvette. A single pulse of 2.5 kV was applied, and the cells were immediately recovered in liquid media and incubated overnight at 37 °C. Positive clones were selected on solid medium containing the 25 μg/mL kanamycin.

### 2.8 Liquid IC_50_ determination

The impact of HTB-04 on *M. bovis* BCG mutant strains over-expressing *Rv0183* was analyzed in liquid media. Using a 96-well plate (flat, black bottom, polystyrene), 1 μL compound (100% DMSO) at 100× desired concentration (0.14–21.8 μM) was aliquoted in triplicate into each well. The different strains were cultured to OD_600nm_ 0.4–0.8 and diluted to 1 × 10^6^ colony-forming units (CFU)/mL, before adding 100 μL to each well. The plate was incubated at 37 °C, 5% CO_2_ for 7 days. 30 μL 0.02% (w/v) resazurin and 12.5 μL 20% (v/v) Tween 80 was added to each well and incubated for a further 24 h. Cell viability was determined by measuring the fluorescence with a λ excitation at 530 nm and λ emission at 590 nm using a POLARstar Omega plate reader. GraphPad Prism was used to fit the data using non-linear regression

### 2.9 Expression and purification of recombinant Rv0183

Recombinant WT BCG_0220 and the mutants BCG_0220-S172P and BCG_0220-VAAQD137D (equivalent to and henceforth referred to as Rv0183 variants as described) were over-expressed in *E. coli* SHuffle T7 (K12) cells and *E. coli* BL21 (DE3) cells, respectively, from the pET28a construct. An overnight culture was used to inoculate 1 L Terrific broth and incubated at 37 °C, 180 rpm, until OD_600nm_ 0.4–06 was reached. The cultures were cooled to 16 °C before protein production was induced using 1 mM IPTG. Cells were cultured overnight at 16 °C before harvesting the following day and the resulting cell pellet was frozen at −20 °C.

The cell pellet was resuspended in 50 mM sodium phosphate, pH 7.5, 0.5 M NaCl and 25 mM imidazole and DNAse and a Complete protease inhibitor cocktail tablet (Roche) were added. Cells were lysed by sonication, 30 s on, 30 s off, for 12 cycles and the insoluble material was pelleted at 14000×*g* for 45 min. The recombinant protein was purified from the clarified lysate using immobilized metal affinity chromatography (IMAC) using a 1 mL nickel-charged IMAC column. The column was washed and the recombinant protein was eluted using a step gradient of 25, 50, 100, 150, 200, 300 and 1000 mM imidazole. Here, fractions containing the purified Rv0183-S172P and Rv0183-VAAQD137D recombinant proteins were dialyzed into 50 mM Tris pH 7.5, 500 mM NaCl, 10% (v/v) glycerol. The WT Rv0183 recombinant protein was desalted into 50 mM Tris pH 7.5, 10% (v/v) glycerol and was further purified by anion exchange using a 1 mL Q HP anion exchange column with an increasing NaCl gradient. Purified proteins were stored at −20 °C.

### 2.10 TLC analysis of Rv0183 activity

Each reaction was performed in 50 mM Tris, pH 7.5, 200 mM NaCl, with a final volume of 50 μL. 10 μM Rv0183 (WT and mutants) was incubated with 5 μg compound (HTB-03, HTB-04, rhein, diacerein) with a final DMSO concentration of 2%. Enzyme-free and compound-free controls were also performed. The reactions were incubated at 37 °C for 1 h before being dried by vacuum centrifugation. The samples were resuspended in methanol and loaded onto a TLC plate (silica gel 60) and developed in either 40:10:1 (v/v) chloroform:methanol:ammonia (for separation of rhein and diacerein) or 80:20 pet-ether (60–80):ethyl acetate (for separation of HTB-03 and HTB-04). Compounds were visualised using UV radiation.

## 3 Results

### 3.1 Anti-mycobacterial activities, toxicity and stability of anthraquinone analogues

To investigate the therapeutic potential of anthraquinone analogues as *Mtb* inhibitors we designed and synthesized two series of esters (Fig. 1) through esterification of rhein and diacetyl rhein (diacerein, a marketed drug for the treatment of osteoarthritis) with representative different chain length alcohols, such as methanol, 2,6-dimethylcyclohexanol and 4-chloro-1-butanol. All six compounds together with rhein and diacerein (Fig. 1) were evaluated for their anti-mycobacterial activities against *Mtb* H_37_Rv using the Microplate Alamar Blue Assay (MABA) and further tested for their cytotoxicity against a mammalian cell line (Vero cells). As shown in Fig. 1, HTB compounds displayed potent *in vitro* anti-mycobacterial activities with MICs in the range from 2.56 to 7.31 μM compared to their parent compounds rhein and diacerein. Interestingly, compounds esterified from rhein exhibited low cytotoxicity with IC_50_ > 162 μM, whereas compounds esterified from diacerein displayed some toxicity with IC_50_ in the range of 15.48 to 83.99 μM.

To identify the metabolic liabilities of the novel HTB compounds, three compounds (HTB-03, HTB-04 and HTB-05) with more potent anti-mycobacterial activities were investigated for their stability in mouse liver microsome (MLM) and human liver microsome (HLM). As summarized in Table 2, Table 3, compounds HTB-03 and HTB-04 with the flexible side chain R_1_ exhibited good to excellent stability in MLM or HLM, while the compound with rigid cyclic side chain R_1_ (HTB-05) showed a relatively low microsomal stability both in MLM and HLM, exemplified by the substrate remaining of HTB-05 in MLM as 42.9% while the substrate remaining of HTB-03 and HTB-04 as 66.3% and 79.8% respectively. Therefore, compounds HTB-03 and HTB-04 were further tested against two drug-resistant *Mtb* clinical isolates. The two compounds displayed potent *in vitro* activity not only against drug-susceptible but also drug-resistant *Mtb* clinical strains (Table 2). These compounds could be promising novel scaffolds for TB drug discovery against drug-resistant strains.

**Table 2:** Anti-tubercular activity and liver microsome stability of the selected compounds. The MICs of the selected HTB compounds against different *Mtb* strains are shown. *^a^Mtb* resistant to isoniazid (INH), rifampicin (RFP), streptomycin (SM), ethambutol (EMB), rifapentine (RFT), rifabutin (RFB) and paza-aminosalicylate (PAS). *^b^Mtb* resistant to INH, EMB, RFP, RFB, RFT, amikacin (AMK) and capreomycin (CPM). Compound stability was analyzed in liver microsomes. *^c^*Substrate concentrations were determined in incubations with NADPH after 30 min and normalized to concentrations at time zero. MIC, minimum inhibitory concentration; MLM, mouse liver microsome; HLM, human liver microsome.

Compounds	MIC (μM)			IC_50_ (Vero) (μM)	Substrate remaining (%)^c^	
	H_37_Rv	12611^a^	14231^b^		MLM	HLM
HTB-03	2.56	2.64	2.40	>170.77	66.3	92.8
HTB-04	3.55	3.66	2.04	83.99	79.8	82.4
HTB-05	3.90	–	–	>162.26	42.9	67.8
INH	0.26	141.97	>291.67	–	–	–

**Table 3:** Whole genome sequencing results of *M. bovis* BCG spontaneous resistant mutants raised against HTB-03 and HTB-04. High frequency single nucleotide polymorphisms resulting in changes in the amino acid sequence are reported. Genomic positions of the SNP are relative to *M. bovis* BCG strain Pasteur 1173P2. The base changes (deletions, insertions or substitutions) are capitalized. The amino acid changes are documented, where *^1^, *^2^, *^3^ and *^4^ represent the generation of a stop codon, amino acid deletions, insertion of a base leading to an amino acid change and a subsequent frameshift, and amino acid insertions. Spontaneous resistant mutants are numbered and the MIC at which they were generated is shown. Dots represent areas where sequence coverage was not sufficient for analysis. Blank spaces indicate the absence of a base change relative to the reference strain.

Gene	Genome position of SNP	Codon change	Amino acid changes	Frequency of SNP	HTB-03 Mutants					HTB-04 Mutants		
					50 × MIC		100 × MIC			20 × MIC		
					1	2	1	2	3	1	2	3
*BCG_0220*	244,283	taT/taG	Y99*^1^	98.86				G				
	244,393	GTGGCGGCACAGgac/gac	VAAQD137D*^2^	97.09	GTGGCGGCACAG		GTGGCGGCACAG			GTGGCGGCACAG		GTGGCGGCACAG
	244,500	Tct/Cct	S172P	100							C	
*BCG_0404c*	475,202	Gcc/Acc	A143T	100	A	A	A		A	A		A
Non-coding region	1,344,671	C/G	–	100	G	G	G	G	G	G	G	G
	1,344,672	G/C	–	100	C	C	C	C	C	C	C	C
*PE_PGRS28*	1,661,298	Gcc/Acc	A651T	100	.	A	.	.	.	A	.	.
	1,661,300	gTc/gGc	V650G	100	.	G	.	.	.	G	.	.
	1,661,303	aTc/aAc	I649N	100	.	A	.	.	.	A	.	.
	1,661,304	Atc/Gtc	I649V	100	.	G	.	.	.	G	.	.
	1,661,306	aTc/aGc	I648S	100	.	G	.	.	.	G	.	.
	1,661,307	Atc/Gtc	I648V	100	.	G	.	.	.	G	.	.
	1,661,309	gAc/gGc	D647G	100	.	G	.	.	.	G	.	.
*pks12*	2,282,655	Ggt/Agt	G2662S	100								
	2,282,656	tgG/tgA	W2661*^1^	100								
*proB*	2,702,724	Gcc/Tcc	A232S	100							T	
*fadD28*	3,237,397	ttt/ttAt	F25L*^3^	100	TA	TA	TA		TA	TA		TA
*BCG_3499c*	3,833,488	gcc/gcGGCc	A433AA*^4^	100	GGC	GGC	GGC	GGC	GGC	GGC	GGC	
*cut3*	3,854,969	ggc/gGTCgc	G261GR*^4^	100	GTC	GTC	GTC	GTC	GTC	GTC		GTC
*PE_PGRS53*	3,907,860	Aac/Gac	N599D	100	G	G	G	G	G	G	G	G
*PE_PGRS57*	3,927,720	Acc/Gcc	T598A	100	G	.	.	.	.	G	.	.
*BCG_3742*	4,098,093	gaC/gaA	D188E	100	A	A	A		A	A		A
*glpK*	4,111,717	ttG/ttC	L385F	100	C	C	C		C	C		C

### 3.2 Efforts to identify the molecular target of the anthraquinone analogues through the generation of resistant isolates

In drug discovery, elucidating the molecular target of an inhibitory compound is a primary step, and can provide valuable information to direct medicinal chemistry efforts to improve compound efficacy and toxicity for the progression into clinical drug candidates. Without prior information of the target, whole genome sequencing of spontaneous resistant mutants generated against an inhibitor is a validated approach to begin de-convoluting its molecular mode of action (Abrahams et al., 2016, Abrahams et al., 2012, Abrahams et al., 2017, Gurcha et al., 2014). This methodology was employed to probe the target of HTB-03 and HTB-04, and ultimately rhein, which could open up new opportunities for discovering novel anti-TB drug candidates through structural modification.

Given the minimal inhibitory concentrations (MICs) of HTB-03, HTB-04 and rhein determined in *Mtb* (Fig. 1; 2.56, 3.55 and 106.26 μM, respectively), spontaneous resistant mutants were generated in the *Mtb* model organism, *M. bovis* BCG, at 50× and 100× MIC of HTB-03 and 20× MIC of HTB-04 with frequencies of resistance of 83 × 10^-8^, 100 × 10^-8^ and 85 × 10^-8^, respectively. Following confirmation of the resistant phenotype of five mutants raised against HTB-03 and three mutants raised against HTB-04, the gDNA was extracted and the whole genome was sequenced. Through comparison to the wild-type (WT) *M. bovis* BCG reference strain (Genbank accession number NC_008769.1), a number of statistically relevant SNPs were identified (Table 3). Three out of the five mutants generated against HTB-03 and all three mutants generated against HTB-04 had mutations in the gene *BCG_0220*, suggesting a role in the resistance mechanism. It is noteworthy that there are a number of SNPs in other genes with high frequencies of occurrence within the population sequenced. These genes were not considered to be involved in the resistance phenotype either because they are commonly observed during *in vitro* spontaneous resistant mutant generation, or because they occurred only in a minority of the mutants sequenced. The *BCG_0220* gene has 100% sequence identity to *Rv0183*, which has been characterized as a monoglyceride lipase (Cotes et al., 2007). Although classified by Himar1-based transposon mutagenesis as non-essential (Sassetti et al., 2003), *Rv0183* is highly conserved and present in the minimal genome of *Mycobacterium leprae*. Additionally, its activity is maintained in dormant *Mtb* (Tallman et al., 2016), suggesting its essential role in the survival and pathogenicity of *Mtb*, warranting further investigation for its role in resistance against the HTB-03 and HTB-04 compounds.

### 3.3 Over-expression of Rv0183 in HTB resistant strains increases sensitivity to the anthraquinone analogues

**Fig. 2:**
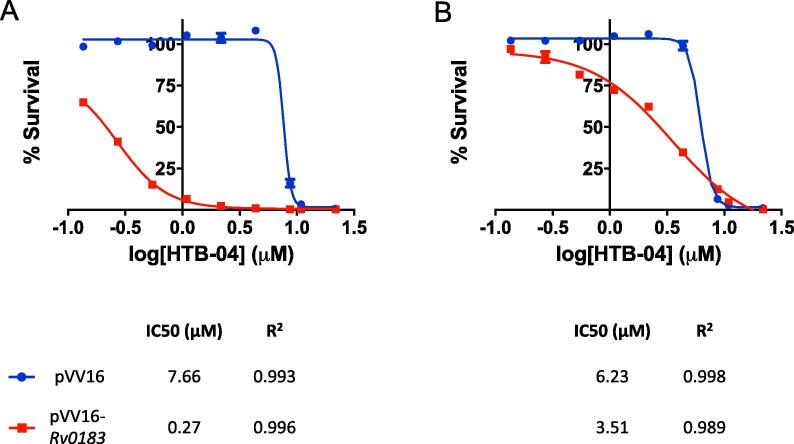
**The impact on survival of HTB resistant mutants over-expressing WT Rv0183**. The mycobacterial expression constructs pVV16 (blue) and pVV16-*Rv0183* (red) were electroporated into the HTB-04 resistant mutants (A) 1 (BCG_0220-VAAQD137D) and (B) 2 (BCG_0220-S172P) and the percentage survival was analyzed using increasing concentrations of HTB-04. Each point shows the mean with bars representing the standard error based on triplicate data. GraphPad Prism was used to fit the data using non-linear regression. IC_50_ and the corresponding R^2^ values are shown. (For interpretation of the references to colour in this figure legend, the reader is referred to the web version of this article.)

From the WGS results, the SNPs in *BCG_0220* are codon deletions, STOP mutations or changes to a conserved residue. These mutations suggest that the reduction or inactivation of BCG_0220 activity results in resistance and from this we can infer that Rv0183 is not the target of the HTB compounds. To assess the involvement of Rv0183 in the resistance mechanism, the impact on the MIC of HTB-04 was investigated using two resistant mutant strains (HTB-04 mutant 1, BCG_0220-VAAQD137D and HTB-04 mutant 2, BCG_0220-S172P (mutation of a conserved residue (Cotes et al., 2007)); mutant nomenclature taken from Table 3). The mycobacterial expression vectors, pVV16 (empty vector) and pVV16-*Rv0183* (over-expressing WT Rv0183 (BCG_0220)) were electroporated into the mutant strains and the liquid MIC was analyzed as shown in Fig. 2. In both mutant strains, the over-expression of Rv0183 markedly reduced the IC_50_ of HTB-04 compared to the empty vectors strain (mutant 1, 7.66 to 0.27 μM; mutant 2, 6.23 to 3.51 μM), indicating a restoration of sensitivity and suggesting a role of the mutated forms of Rv0183 in the resistance mechanism.

### 3.4 Rv0183 hydrolyzes the chlorinated acyl chain of the anthraquinone analogues

**Fig. 3:**
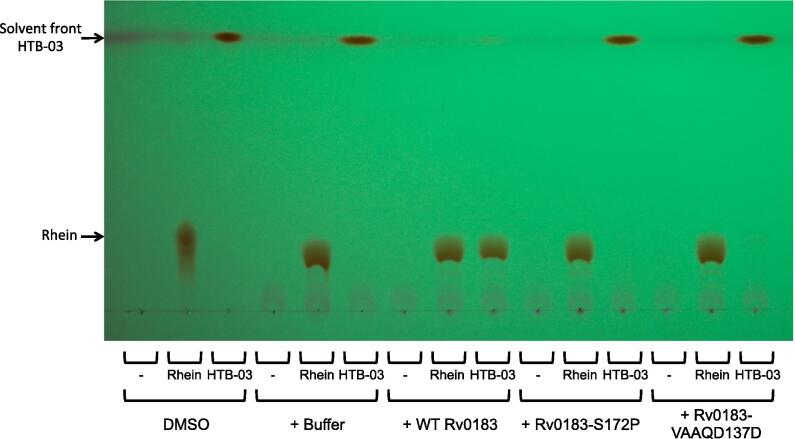
**TLC analysis of HTB-03 compound modification by Rv0183**. Rv0183, WT and mutant variants, were incubated with Rhein and HTB-03 and the samples were analyzed by TLC (40:10:1 (v/v) chloroform:methanol:ammonia) and exposed under UV light. For reference, compounds in DMSO were loaded directly onto the TLC and were not subjected to the assay conditions. Compound only in buffer and enzyme only incubations were used as controls. The solvent front and the positions of HTB-03 and rhein are indicated.

After establishing the involvement of Rv0183 in the resistance to HTB compounds, the mechanism was investigated. Relating the structure of the compounds with the characterized activity of Rv0183, it was plausible that the enzyme hydrolyzed the ester linkage, releasing the chlorinated carbon chain. To validate this hypothesis, recombinant WT BCG_0220, BCG_0220-S172P and BCG_0220-VAAQD137D (equivalent to and henceforth referred to as Rv0183 variants as described) were incubated with HTB-03 and HTB-04 and the compounds were analyzed by thin layer chromatography (TLC). As controls, the migration of the HTB-03 derivative, rhein, and the HTB-04 derivative, diacerein, (without the chlorinated carbon chain) were analyzed alongside the HTB compounds. In the absence of enzyme, HTB-03 and HTB-04 migrated with the solvent front (Fig. 3, Supplementary Fig. 1A). Fig. 3 shows that upon incubation with WT Rv0183, HTB-03 was converted to rhein. However, the mutants Rv0183-VAAQD137D and Rv0183-S172P showed minimal and no activity with HTB-03, respectively. The enzymes had no apparent activity on rhein. The activities of the enzymes with HTB-04 were inconclusive (Supplementary Fig. 1). In addition to the limited solubility of HTB-04, it was evident that the R_2_ acyl groups on diacerein were hydrolyzed in the buffer system used, leading to the production of rhein (Supplementary Fig. 1A). This was also apparent for the R_2_ acyl groups on HTB-04, where partial degradation of the compound led to the generation of various products, one of which coincided with HTB-03 (Supplementary Fig. 1B). Upon the incubation of HTB-04 with Rv0183, a band representing rhein appeared. Despite this apparent activity, it was not possible to confirm whether Rv0183-dependent hydrolysis of the chlorinated carbon tail occurred before or after the enzyme-independent hydrolysis of the R_2_ acyl groups on the diacerein moiety. Also, due to the instability of diacerein in the assay conditions, it was also not possible to determine whether Rv0183 was able to hydrolyze the acyl groups in addition to the spontaneous degradation. Regardless of limited information gained from the enzyme activity with HTB-04, the hydrolysis of the chlorinated acyl chain of HTB-03 implicates rhein as the active moiety of the HTB compounds.

## 4 Discussion

### 4.1 Derivatives of rhein as suitable compounds to treat *Mtb*

The natural product, rhein, has a number of pharmacological activities, and its known antimicrobial properties directed this investigation as to whether rhein derivatives could be used to treat *Mtb*. It has, however, previously been demonstrated that rhein does not inhibit *Mtb* (Gajadeera et al., 2015). Mycobacteria possess an extensive lipophilic mycobacterial cell wall, and it is this feature that could prevent entry of rhein into the cell and imparting its effect. Therefore, we envisaged that optimizing the chemical structure to one that was more lipophilic, could aid entry into the cell. From this, HTB-03 and HTB-04 (derivatives of rhein and diacerein, respectively), which have flexible chlorinated carbon chains, were synthesized. Both compounds exhibit anti-tubercular activities as well as low toxicity and good liver microsome stability. This preliminary result revealed that there are more opportunities to identify promising leads for the anti-TB drug discovery with novel scaffolds.

### 4.2 Mutations in the lipase, Rv0183, conferred resistance to the HTB compounds

To further progress the HTB compounds, their mode of action was investigated, firstly via WGS of resistant mutants. Although the target was not elucidated, we have identified that mutations in *BCG_0220* are responsible for resistance against the HTB compounds. From this, we have deduced that the HTB compounds are prodrugs, requiring activation by the *Mtb* homologue *Rv0183*. Resistance associated with prodrug activation is a common problem associated with the first-line drug in TB treatment, isoniazid, where mutations in *katG* prevent compound activation and the generation of the isoniazid-NADH adduct, which exhibits anti-tubercular activity. In clinical MDR isolates and INH-resistant strains generated within the laboratory, the frequency of mutations relating to the actual target, *inhA*, are low in comparison to *katG* (Jagielski et al., 2014, Tseng et al., 2015), indicating the potential difficulties with identifying the target of the HTB compounds through whole genome sequencing of resistant isolates. In this regard, we attempted to generate spontaneous resistant mutants against the hypothesized active moiety of the HTB compounds, rhein, but were unsuccessful despite testing a broad range of concentrations.

### 4.3 The role of Rv0183 in prodrug activation

**Fig. 4:**
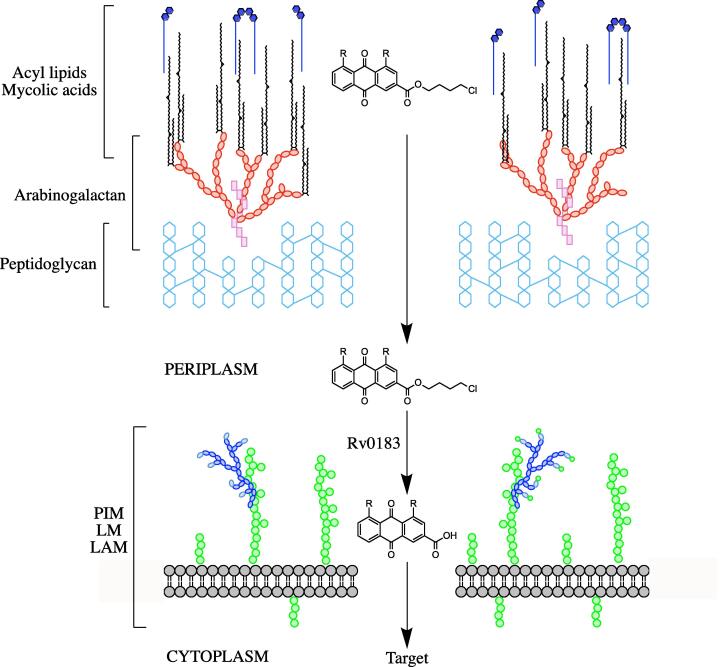
**Model of HTB entry and activation into the mycobacterial cell**. The HTB-03 or HTB-04 compound is shown to traverse the lipid layer, exploiting the lipophilic chain. In the periplasm, Rv0183 hydrolyzes the ester linkage, releasing 4-chlorobutan-1-ol. We propose that rhein (or diacerein) can be imported through the cytoplasmic membrane into the cell to exert its effect. R, —OH (HTB-03) or —COOH (HTB-04). PIM, phosphatidyl-*myo*-inositol mannoside; LM, lipomannan; LAM, lipoarabinomannan.

In this work, we have discovered that Rv0183 restores sensitivity to HTB mutants in a whole cell assay, and that recombinant Rv0183 hydrolyzes the ester linkage on the rhein derivative HTB-03, *in vitro*. Unfortunately, we were unable to establish enzyme activity on the diacerein derivative HTB-04 due to compound insolubility and the apparent instability of the acyl groups in our buffer system. Therefore, it is not possible to conclude whether diacerein can function as the active molecule, or whether further modifications by other lipases are necessary prior to or post Rv0183 activity. The higher MIC of HTB-04 compared to HTB-03 could indicate the latter is plausible. Interestingly, due to the high MIC exhibited by rhein in *Mtb*, the chlorinated 4-carbon chain of the HTB compounds must contribute to their potency. Previous localization studies have revealed that Rv0183 exists in the cell wall and extracellular space (Cotes et al., 2007). From these considerations, we propose a model in which the HTB compounds traverse the lipid rich mycobacterial cell wall by exploiting the carbon chain, where the compounds interact with Rv0183 in the periplasmic space, releasing rhein, which can pass through the cytoplasmic membrane, where it can be further modified, if necessary, and reach the target (depicted in Fig. 4). In this model, the activity of Rv0183 would have to be regulated; activity exterior to the cell wall would convert the compounds into rhein or similar, which is essentially inactive against *Mtb*. It has been suggested that Rv0183 is bound to large particles on the outside of the cell wall (Cotes et al., 2007), the role of which could control the activity of the lipase.

### 4.4 Rv0183 as a direct target for anti-tubercular drugs

The activity of lipases is important during the survival and pathogenicity of *Mtb*, where lipid metabolism is key to providing a carbon source during infection, dormancy and reactivation. It has recently been established that Rv0183, along with CaeA (esterase and protease) and Rv1683 (long-chain acyl-CoA synthase and lipase), are active during all stages of TB disease (Tallman et al., 2016). Furthermore, disruption of the ortholog of Rv0183 in *Mycobacterium smegmatis* has been shown to impact drug susceptibility (Dhouib et al., 2010). These features make these enzymes, along with their accessible location, suitable targets for anti-tubercular drugs, which are currently being pursued (Aschauer et al., 2018, Point et al., 2013, Point et al., 2012, Saravanan et al., 2012). As we have proposed that Rv0183 is involved in the activation of the HTB compounds, inhibition of this enzyme would be undesirable in the context of the potential treatment with compounds of this class. Dormant *Mtb* has a limited enzymatic activity, and enzymes that remain active are expected to be essential to sustain survival, especially during latency. In this regard, inactivating mutations in Rv0183, although causing resistance against the HTB compounds, would likely be prohibitory for survival. Additionally, due to the activity of Rv0183 during dormancy, the activated HTB compounds maintain the potential (target dependent) to inhibit latent *Mtb* infections. To conclude, the HTB compounds and other rhein derivatives could be conceivably progressed into clinical drug candidates, and this will be further advanced by the elucidation of the molecular target.

## 5 Author statements

### 5.1 Ethics statement

All experiments were approved by the University of Birmingham, Institute of Materia Medica, and Beijing Tuberculosis and Thoracic Tumor Research Institute ethical  committees where required and there are no ethical issues to  report.

## CRediT authorship contribution statement

**Katherine A. Abrahams:** Conceptualization, Methodology, Validation, Formal analysis, Investigation, Writing - original draft, Writing - review & editing, Visualization. **Wei Hu:** Conceptualization, Methodology, Validation, Investigation. **Gang Li:** Conceptualization, Methodology, Validation, Formal analysis, Investigation. **Yu Lu:** Conceptualization, Methodology, Validation, Investigation. **Emily J. Richardson:** Formal analysis, Investigation. **Nicholas J. Loman:** Formal analysis, Investigation. **Haihong Huang:** Conceptualization, Methodology, Validation, Formal analysis, Investigation, Resources, Writing - original draft, Writing - review & editing, Visualization, Supervision, Project administration. **Gurdyal S. Besra:** Conceptualization, Methodology, Validation, Formal analysis, Investigation, Resources, Writing - original draft, Writing - review & editing, Visualization, Supervision, Project administration.

## Declaration of Competing Interest

The authors declare that they have no known competing financial interests or personal relationships that could have appeared to influence the work reported in this paper.
