# Supplementary material for: Anti-tubercular derivatives of rhein require activation by the monoglyceride lipase Rv0183
Source: Cell Surf. 2020 Apr 21;6:100040. doi: 10.1016/j.tcsw.2020.100040 (PMC7389528; doi:10.1016/j.tcsw.2020.100040)
Supplement: Supplementary data 1 [file mmc1.docx]

**Supplementary Material**

**Anti-tubercular derivatives of rhein require activation by the monoglyceride lipase Rv0183**

Katherine A. Abrahams^1^, Wei Hu^2^, Gang Li^2^, Yu Lu^3^, Emily J. Richardson^4^, Nicholas J. Loman^1^, Haihong Huang^2*^, and Gurdyal S. Besra^1*^

^1^Institute of Microbiology and Infection, School of Biosciences, University of Birmingham, Edgbaston, Birmingham B15 2TT, UK

^2^State Key Laboratory of Bioactive Substances and Function of Natural Medicine, Beijing Key Laboratory of Active Substance Discovery and Druggability Evaluation, Institute of Materia Medica, Peking Union Medical College and Chinese Academy of Medical Sciences, 1 Xian Nong Tan Street, Beijing 100050, China

^3^Beijing Key Laboratory of Drug Resistance Tuberculosis Research, Department of Pharmacology, Beijing Tuberculosis and Thoracic Tumor Research Institute, Beijing Chest Hospital, Capital Medical University, 97 Ma Chang Street, Beijing 101149, China

^4^MicrobesNG, Units 1-2 First Floor, The BioHub, Birmingham Research Park, 97 Vincent Drive, Birmingham, B15 2SQ, UK

^*^E-mail for correspondence: g.besra@bham.ac.uk (TEL: +00 44 121 415 8125; FAX +00 44 121 414 5925)

^*^E-mail for correspondence: joyce@imm.ac.cn (TEL: +86 10 63165244; FAX +86 10 63165244)

**Supplementary** **Methods**

The target compounds were synthesized following the procedure as outlined in **Scheme 1**. The rhein or diacerein was treated with thionyl chloride under reflux condition to afford the acyl chloride intermediate. After removing the remaining thionyl chloride, the residue was treated with the corresponding alcohol in CH_2_Cl_2_ in the presence of DMAP at room temperature to give the target HTB compounds after purification by column chromatography. The structures of all new target compounds were characterized by ^1^H NMR and MS.

**Scheme 1: Synthesis of the target compounds*^a^***

*^a^*Reagents and conditions: i) SOCl_2_, CH_2_Cl_2_, reflux, 4 h; ii) R_1_OH, DMAP, CH_2_Cl_2_, rt, 3-6 h, 22-67%.

**General experimental information**

All the solvents and chemicals were obtained from commercial sources and used without further purification. Thin layer chromatography was performed on silica gel plates (GF254) with visualization of components by UV light (254 nm) or exposure to I_2_. Column chromatography was carried out on silica gel (300-400 mesh). The structural identities of the prepared compounds were confirmed by ^1^H NMR and mass spectrometry. ^1^H NMR spectra were obtained on Varian Mercury-400 at 400 MHz. Chemical shifts (δ) values were referenced to the residual solvent peak and reported in ppm and all coupling constant (*J*) values were given in Hz. CDCl_3_ were used as the standard NMR solvents. The following multiplicity abbreviations are used: (s) singlet, (d) doublet, (t) triplet, (q) quartet, (m) multiplet, and (brs) broad. ESI-MS data were measured on Thermo Exactive Orbitrap Plus spectrometer.

**NMR and MS data of the HTB compounds**

*Methyl 4,5-dihydroxy-9,10-dioxo-9,10-dihydroanthracene-2-carboxylate (HTB-01).* Dark yellow solid; yield 67%. ^1^H NMR (400 MHz, CDCl_3_) δ: 12.03 (s, 1H), 11.97 (s, 1H), 8.43 (d, *J* = 1.6 Hz, 1H), 7.94 (d, *J* = 1.6 Hz, 1H), 7.88 (dd, *J_1_* = 8.8 Hz, *J_2_* = 1.2 Hz, 1H), 7.73 (t, *J* = 8.0 Hz, 1H), 7.34 (dd, *J_1_* = 8.8 Hz, *J_2_* = 1.2 Hz, 1H), 4.00 (s, 3H). MS (ESI) *m/z* 299.1 (M+H)^+^.

*3-(Methoxycarbonyl)-9,10-dioxo-9,10-dihydroanthracene-1,8-diyl diacetate (HTB-02).* Light yellow solid; yield 66%. ^1^H NMR (400 MHz, CDCl_3_) δ: 8.82 (d, *J* = 1.8 Hz, 1H), 8.25 (dd, *J_1_* = 8.0 Hz, *J_2_* = 1.2 Hz, 1H), 8.04 (d, *J* = 1.8 Hz, 1H), 7.79 (t, *J* = 8.0 Hz, 1H), 7.43 (dd, *J_1_* = 8.0 Hz, *J_2_* = 1.2 Hz, 1H), 3.99 (s, 3H), 2.45 (s, 3H), 2.44 (s, 3H). MS (ESI) *m/z* 405.1 (M+Na)^+^.

*4-Chlorobutyl 4,5-dihydroxy-9,10-dioxo-9,10-dihydroanthracene-2-carboxylate (HTB-03).* Dark yellow solid; yield 37%. ^1^H NMR (400 MHz, CDCl_3_) δ: 12.06 (s, 1H), 11.99 (s, 1H), 8.43 (d, *J* = 1.6 Hz, 1H), 7.95 (d, *J* = 1.6 Hz, 1H), 7.89 (dd, *J_1_* = 7.6 Hz, *J_2_* = 1.2 Hz, 1H), 7.45 (t, *J* = 8.0 Hz, 1H), 7.35 (dd, *J_1_* = 8.4 Hz, *J_2_* = 1.2 Hz, 1H), 4.45-4.42 (m, 2H), 3.65-3.63 (m, 2H), 2.00-1.97 (m, 4H). MS (ESI) *m/z* 375.1 (M+H)^+^.

*3-((4-Chlorobutoxy)carbonyl)-9,10-dioxo-9,10-dihydroanthracene-1,8-diyl diacetate (HTB-04).* Light yellow solid; yield 43%. ^1^H NMR (400 MHz, CDCl_3_) δ: 8.82 (d, *J* = 1.8 Hz, 1H), 8.26 (dd, *J_1_* = 8.0 Hz, *J_2_* = 1.2 Hz, 1H), 8.03 (d, *J* = 1.8 Hz, 1H), 7.81 (t, *J* = 7.8 Hz, 1H), 7.45 (dd, *J_1_* = 8.0 Hz, *J_2_* = 1.2 Hz, 1H), 4.44 (t, *J* = 6.2 Hz, 2H), 3.64 (t, *J* = 6.0 Hz, 2H), 2.47 (s, 3H), 2.46 (s, 3H), 2.00-1.97 (m, 4H). MS (ESI) *m/z* 481.2 (M+Na)^+^.

*2,6-Dimethylcyclohexyl 4,5-dihydroxy-9,10-dioxo-9,10-dihydroanthracene-2-carboxylate (HTB-05).* Dark yellow solid; yield 31%. ^1^H NMR (400 MHz, CDCl_3_) δ: 12.08-12.05 (m, 1H), 12.01-11.99 (m, 1H), 8.46-8.45 (m, 1H), 7.97 (dd, *J_1_* = 9.2 Hz, *J_2_* = 1.2 Hz, 1H), 7.89 (m, 1H), 7.74 (t, *J* = 7.8 Hz, 1H), 7.36-7.34 (m, 1H), 4.92-4.62 (m, 1H), 2.22 (brs, 1H), 2.05 (brs, 1H), 1.83-1.68 (m, 3H), 1.64-1.59 (m, 1H), 1.54 (s, 6H), 1.25-1.15 (m, 2H). MS (ESI) *m/z* 395.1 (M+H)^+^.

*3-(((2,6-Dimethylcyclohexyl)oxy)carbonyl)-9,10-dioxo-9,10-dihydroanthracene-1,8-diyl diacetate (HTB-06).* Light yellow solid; yield 22%.^1^H NMR (400 MHz, CDCl_3_) δ: 8.84-8.83 (m, 1H), 8.27-8.25 (m, 1H), 8.07-8.03 (m, 1H), 7.80 (t, *J* = 8.0 Hz, 1H), 7.44 (dd, *J_1_* = 8.0 Hz, *J_2_* = 1.2 Hz, 1H), 4.93-4.90 (m, 1H), 2.48-2.47 (m, 3H), 2.46 (s, 3H), 2.22 (brs, 1H), 2.07-2.05 (m, 1H), 1.83-1.68 (m, 3H), 1.64-1.57 (m, 1H), 1.54 (s, 6H), 1.28-1.16 (m, 2H). MS (ESI) *m/z* 501.2 (M+Na)^+^.

**Supplementary Figures**

**Supplementary Figure 1: TLC analysis of Rv0183 activity on HTB-04.** The activity of Rv0183 on the diacerein derivative HTB-04 was assessed. Assays were performed at 37°C for 1 h and the effect on the compounds was analyzed by TLC. A) Rv0183 activity on HTB-04. In the absence of enzyme, diacerein degraded to rhein. In the presence of enzyme, HTB-04 partially hydrolyzed to rhein. Due to compound degradation, it was not possible to determine whether the ester groups on the diacerein moiety were hydrolyzed before or after enzyme-dependent hydrolysis of the chlorinated tail of HTB-04. HTB-04 also exhibited limited solubility in the assay conditions, further impacting the analysis of enzyme activity. To separate rhein and diacerein, the TLC was developed in 40:10:1 (v/v) chloroform:methanol:ammonia. B) Degradation analysis of the HTB compounds in the assay conditions used. HTB-03 appeared stable in the assay conditions used, but degradation of HTB-04 was evident by comparison to the DMSO control. To separate HTB-03 and HTB-04, the TLC was developed in 80:20 pet-ether (60-80):ethyl acetate. For reference, compounds in DMSO were loaded directly onto the TLC and were not subjected to the assay conditions.
